# Frequency and Prognostic Value of IDH Mutations in Korean Patients With Cholangiocarcinoma

**DOI:** 10.3389/fonc.2020.01514

**Published:** 2020-08-18

**Authors:** Nah Ihm Kim, Myung-Giun Noh, Jo-Heon Kim, Eun Jeong Won, Yu Jeong Lee, Younghoe Hur, Kyung-Sub Moon, Kyung-Hwa Lee, Jae-Hyuk Lee

**Affiliations:** ^1^Department of Pathology, Chonnam National University Medical School and Hwasun Hospital, Hwasun-gun, South Korea; ^2^Department of Biomedical Science and Engineering, Gwangju Institute of Science and Technology (GIST), Gwangju, South Korea; ^3^Department of Parasitology and Tropical Medicine, Chonnam National University Medical School, Hwasun-gun, South Korea; ^4^Department of Hepatobiliary Pancreas Surgery, Chonnam National University Medical School and Hwasun Hospital, Hwasun-gun, South Korea; ^5^Department of Neurosurgery, Brain Tumor Clinic & Gamma Knife Center, Chonnam National University Hwasun Hospital and Medical School, Hwasun-gun, South Korea

**Keywords:** cholangiocarcinoma, isocitrate dehydrogenase, mutation, high-throughput nucleotide sequencing, survival rate

## Abstract

The molecular profile of cholangiocarcinoma (CC) remains elusive. The prognostic value of isocitrate dehydrogenase (IDH) mutations in CC is controversial, and there have been few relevant studies in Asian populations. In the present study, we investigated the frequency and prognostic significance of IDH mutations in Korean patients with CC. CC specimens were collected from patients who underwent surgical liver resection between 2004 and 2019. Clinical and pathological data were retrospectively reviewed from medical records. Mutational IDH profiling was performed by peptide nucleic acid-mediated PCR clamping in 206 surgical specimens; IDH-mutant samples were confirmed by next-generation sequencing (NGS). Of the 195 patients with CC, six (3.13%) were found to exhibit IDH1 (*n* = 5) or IDH2 (*n* = 1) mutations. Among patients with IDH1 mutations, four had R132C (c.394C>T) and one had R132G (c.394C>G) mutations. One patient had R172W (c.514A>T) mutations in IDH2. All IDH-mutant samples were of intrahepatic origin, and patients with IDH mutations had physiological to low serum levels of carbohydrate antigen 19-9 (CA19-9). No association between IDH mutation status and long-term survival outcomes was observed. The frequency of IDH mutations was considerably lower than the 10–20% reported in previous studies. The frequency and pattern of IDH mutations in CC are likely to vary among patients with different ethnicities. These findings suggest that characterization of the oncogenic mutation profile in different populations is of high clinical importance.

## Introduction

Cholangiocarcinoma (CC) is a heterogeneous group of malignancies, which can be classified as intrahepatic, perihilar, and distal, according to their anatomic location. Several risk factors have been identified for certain types of CC, including chronic viral hepatitis, bile duct stones, and primary sclerosing cholangitis. The incidence rates of CC vary considerably among different geographical locations; its prevalence is markedly higher in Southeast Asia than in other parts of the world, due to liver fluke infestation. Although tumor resection can provide curative treatment of CC, surgery is only available for 10–20% of patients who are diagnosed at early stages. For patients diagnosed with advanced disease, treatment strategies include combination chemotherapy and targeted molecular therapy. However, patients with advanced CC have a poor prognosis, with a median overall survival of <1 year (1, 2).

Several studies have been performed to identify novel molecular targets in CC. A recent study suggested that patients with intrahepatic CC frequently have inactivating mutations in multiple chromatin-remodeling genes, including *BAP1, ARID1A*, and *PBRM1* (3). Additionally, somatic mutations in isocitrate dehydrogenase 1 (*IDH1*) and *IDH2* have been observed in 10–20% of patients with CC (4–7). However, the relationships of *IDH* mutations to clinicopathologic features and prognosis among patients with CC remains controversial (3–9). *IDH* mutations in CC have been associated with long-term improvement, worsening, or no impact (3, 5, 6, 8, 9). Moreover, while some studies showed that *IDH* mutations were associated with poorly differentiated CC and clear-cell histology (7), others showed no association with histological grade (3). Significant differences in *IDH* mutations between certain types of parasite-associated CC have also been reported (10).

However, most studies have focused on *IDH* mutations in intrahepatic CC and have involved non-Asian cohorts. Therefore, the aim of this study was to assess *IDH* mutations in a large cohort of Korean patients with CC at various anatomic locations, including intrahepatic and extrahepatic lesions. Furthermore, this study explored the associations of *IDH* mutations with clinicopathological features and long-term outcomes in patients with resected CC. In addition, this study used next-generation sequencing (NGS) analysis to investigate differences in *IDH* mutation patterns among patients with CC of different ethnicities.

## Materials and Methods

### Patients and Clinicopathological Data

**Figure 1:**
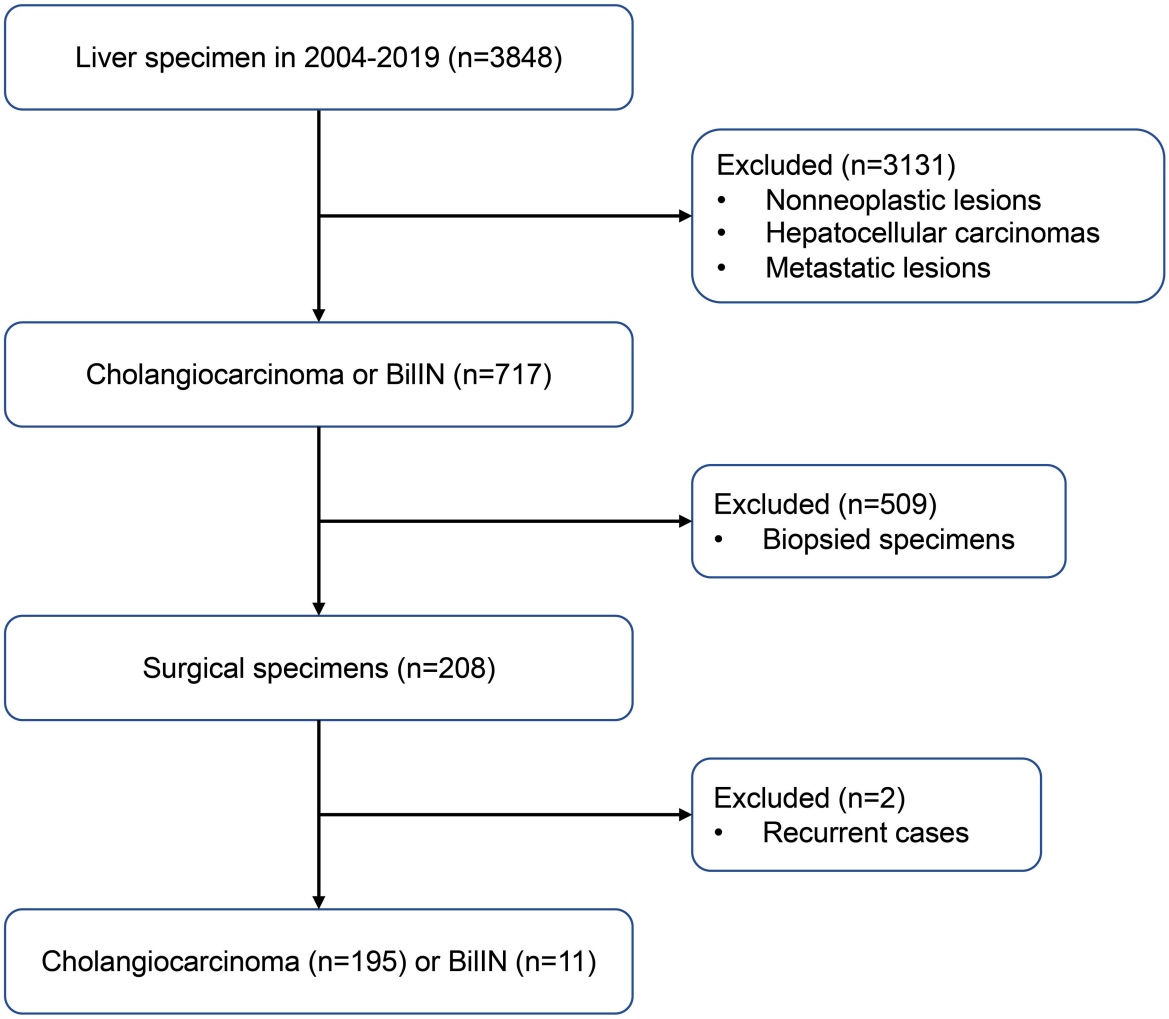
Case selection flow with inclusion and exclusion criteria.

Samples from 195 patients with CC and 11 patients with biliary intraepithelial neoplasia (BilIN) were obtained during surgical resection of the liver at the Chonnam National University Hwasun Hospital between 2004 and 2019. A flow chart of case selection is presented in Figure 1. Clinical data were retrospectively reviewed from the patients' medical records. To investigate relationships between *IDH* mutations and clinicopathological parameters, the following variables were evaluated: age, sex, tumor size, tumor localization, presence of parasites or hepatitis, serum CA 19-9 levels, T stage, lymph node metastasis, distant metastasis, and surgical margin involvement. The pathological stage was determined in accordance with the American Joint Committee on Cancer (AJCC) staging system, 7th edition (11). Surgical margin involvement was defined as the presence of tumor within <1 mm from the excision margin. Disease-free survival (DFS) was calculated from the date of surgery to the date of recurrence, progression, or death. Overall survival (OS) was calculated from the date of surgery until death or the last follow-up visit.

All available tumor tissue slides were independently reviewed by two pathologists (KNI and LKH) to evaluate histopathological features and select representative tissue blocks. Tumors were classified and subtyped according to the World Health Organization classification of tumors of the digestive system (12). The following histopathological features were evaluated: histologic type, histologic grade, presence of mucinous or signet ring cell component, presence of sarcomatoid component, coexistence of BilIN, association with intraductal papillary neoplasm, bile duct stone, periductal inflammation, and presence of cirrhosis. Well-differentiated and moderately differentiated tumors were defined as low grade; poorly differentiated tumors, as well as carcinosarcomas and combined hepatocellular-CCs, were defined as high grade. Periductal inflammation was graded as minimal to mild, or moderate to severe, according to the degree of necroinflammatory activity. This study was approved by the Institutional Review Board of the Chonnam National University Hwasun Hospital (CNUHH-2019-213).

### Detection of IDH Mutations by Peptide Nucleic Acid (PNA)-Mediated Real-Time PCR Clamping

Formalin-fixed paraffin-embedded tissue blocks from 206 patients were used to prepare 10-μm-thick sections. Genomic tumor DNA was extracted using the Maxwell 16 MDx Instrument (Promega, Madison, WI, USA), in accordance with the manufacturer's instructions. The tumor tissues were macro-dissected, in accordance with a previously described method (13). *IDH1* and *IDH2* mutations were analyzed using genomic DNA from 195 patients with CC and 11 patients with BilIN. The extracted genomic DNA was subjected to mutation analysis using the PNAClamp™ *IDH* Mutation detection kit (Panagene, Daejeon, Korea), as previously described (14). The mutation spots of IDH1 and IDH2 that are detected with the PNAClamp™ kit are listed in Supplementary Table 1. The PNA clamping probes were complementary to the wild-type sequence and competitively inhibited the binding of the DNA primers. Consequently, they preferentially amplified the mutant alleles. The reported sensitivity of the PNAClamp™ kit for IDH1/2 mutation testing is 1% with cloned DNA, according to the manufacturer's verification. Real-time PCR reactions were performed using a 7500 Fast Real-Time PCR System (Applied Biosystems, Foster City, CA, USA).

### Next-Generation Sequencing (NGS) Analysis

NGS analysis was performed for the validation of *IDH1* and *IDH2* mutations. Genomic DNA was extracted from tumors after macro-dissection using the Gene-Read™ DNA FFPE Kit (Qiagen, Hilden, Germany), in accordance with the manufacturer's protocol. Targeted sequencing for 90 cancer-related genes was performed using the SureSelect targeted panel (Agilent Technologies, Santa Clara, CA, USA), as previously described (15). The processed libraries were loaded onto the MiSeqDx instrument (Illumina, San Diego, CA, USA), in accordance with the manufacturer's protocol. Sequenced reads were aligned to the human reference genome (GRCh37/hg19) using the BWA-MEM (0.7.15); common germline variants were distinguished from somatic variant candidates in accordance with a previously described method (15).

### Parasite Detection Using PCR

Specimens with parasites were selected after observation under the microscope. Detection and identification of parasites (*Opisthorchis viverrini* and *Clonorchis sinensis*) were achieved using a PCR-based method. Two pairs of species-specific primers were designed to bind to the mitochondrial NADH dehydrogenase subunit 2 (*nad2*) genes of *O. viverrini* and *C. sinensis*. The primer sequences were OV-F (5′-ATG TAG TGT TGG TTG GAG TT-3′) and OV-R (5′-CAC AAT TAC CGC CGT AGC-3′) for *O. viverrini*, and CS-F (5′-GTC TGT TGA GCT TTC TCC T-3′) and CS-R (5′-TAA AGA CCC TGG AAA CGA GAT-3′) for *C. sinensis*. The PCR mixture contained 10 × reaction buffer, 10 mM dNTPs Mixture, Prime Taq DNA polymerase, and each of the four primers (OV-F, OV-R, CS-F, and CS-R), in a total reaction volume of 50 μL. The PCR cycling included one cycle at 95°C for 10 min, followed by 45 cycles of 95°C for 10 s, 55°C for 8 s, and 72°C for 15 s. PCR products were confirmed by sequencing using the same primers.

### Immunohistochemistry

Immunohistochemistry for CD56 was performed on tissues found to exhibit *IDH* mutations. Tissue sections (3 μm thick) were prepared from paraffin-embedded tissue blocks. Tissue slides were subjected to immunohistochemistry staining using an automated immunostainer (Bond-MaX DC2002, Leica Biosystems, Bannockburn, IL, USA). Tissue sections were pretreated with bond epitope retrieval solution 1 (containing citrate buffer, pH 6.0), followed by incubation with CD56 antibody (1:400 dilution; cat. no.: M7304, DAKO, Glostrup, Denmark). Unstained tissues served as negative controls. Immunostained tissues were evaluated independently by two pathologists (KNI and LKH).

### Statistical Analysis

Statistical analysis was performed using SPSS Statistics, version 25.0 (IBM Corp., Armonk, NY, USA) for Windows. To evaluate relationships between *IDH* mutations and clinicopathological parameters, the Pearson chi-squared test or Wilcoxon signed-rank test was used as appropriate. The effects of individual variables on survival were determined by univariate and multivariate analyses. For multivariate analysis, independent prognostic factors were determined using Cox proportional hazards models. Survival rates were calculated using the Kaplan–Meier method, and survival curves were compared using the log-rank test. *P* < 0.05 were considered statistically significant.

## Results

### Clinical Characteristics of Patients With CC

This study involved 195 patients with CC; 138 (70.8%) were men, and the remaining 57 (29.2%) were women. The mean age of patients at diagnosis was 63.1 years (range, 35–80 years). The mean tumor size was 4.38 cm. The majority of tumors were located in the intrahepatic area (168/195; 86.2%); adenocarcinoma (168/195; 86.2%) was the most common histologic type. T2 was the most common stage (81/195, 41.5%), according to the AJCC criteria. Forty-six patients had lymph node metastasis, while four exhibited distant metastasis at the time of diagnosis. Parasites were detected in eight patients by histological examination or gross detection in the operating field. Further PCR analysis using genomic DNA from the tumors and organism-specific primers did not reveal *O. viverrini* or *C. sinensis* infestations in other patients. The clinicopathological features of the patients in this study are summarized in Table 1.

**Table 1:** Associations between *IDH* mutations and clinicopathologic variables.

**Clinicopathologic variables**		**No**.	**IDH mutation**		***P*-value**
			**Present**	**Absent**	
Age (mean, 63.1 years)	≤ 60 years	60	1	59	0.447
	>60 years	135	5	130	
Sex	Male	138	5	133	0.492
	Female	57	1	56	
Size	≤ 4 cm	111	1	110	0.043
	>4 cm	84	5	79	
Location	Intrahepatic	168	6	162	0.319
	Extrahepatic	27	0	27	
Histological type	Adenocarcinoma	168	6	162	0.403
	Carcinosarcoma	2	0	2	
	Combined HCC/CC	25	0	25	
Histological grade	Low-grade	131	6	125	0.082
	High-grade	64	0	64	
Parasite (*Clonorchis sinensis*)	Absent	187	6	181	0.607
	Present	8	0	8	
Hepatitis (HBV or HCV)	Absent	146	6	140	0.149
	Present	49	0	49	
CA19-9	Normal (≤ 39)	56	5	51	0.005
	Elevated (>39)	84	0	84	
T stage	Low (T1 or T2)	128	5	123	0.354
	High (T3 or T4)	67	1	66	
Lymph node mets	Absent	149	5	144	0.685
	Present	46	1	45	
Distant mets	Absent	191	6	185	0.719
	Present	4	0	4	
Surgical margin status	Uninvolved	152	5	147	0.747
	Involved	43	1	42	

*IDH, isocitrate dehydrogenase; CC, cholangiocarcinoma; HCC, hepatocellular-cholangiocarcinoma; HBV, hepatitis B virus; HCV, hepatitis C virus*.

### Relationships Between IDH Mutations and Clinicopathological Features

**Figure 2:**
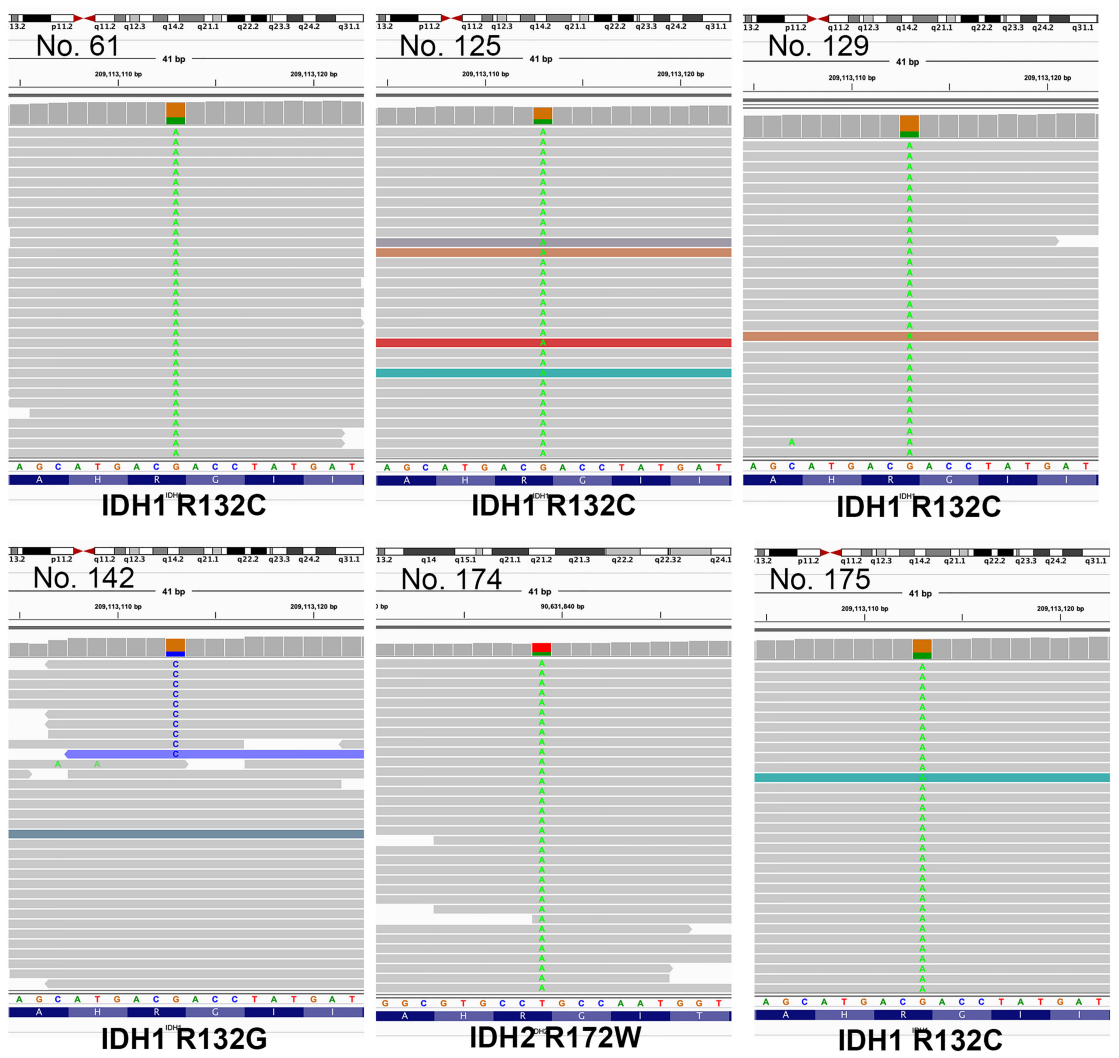
Illustration of mutations identified by next-generation sequencing (NGS), visualized in Integrative Genome Viewer (IGV). Patients 61, 125, 129, and 175 had mutations in codon 132 of *IDH1* (R132C). Patient 142 had R132G mutations, while patient 172 had *IDH2* mutations in codon 172 (R172W).

**Figure 3:**
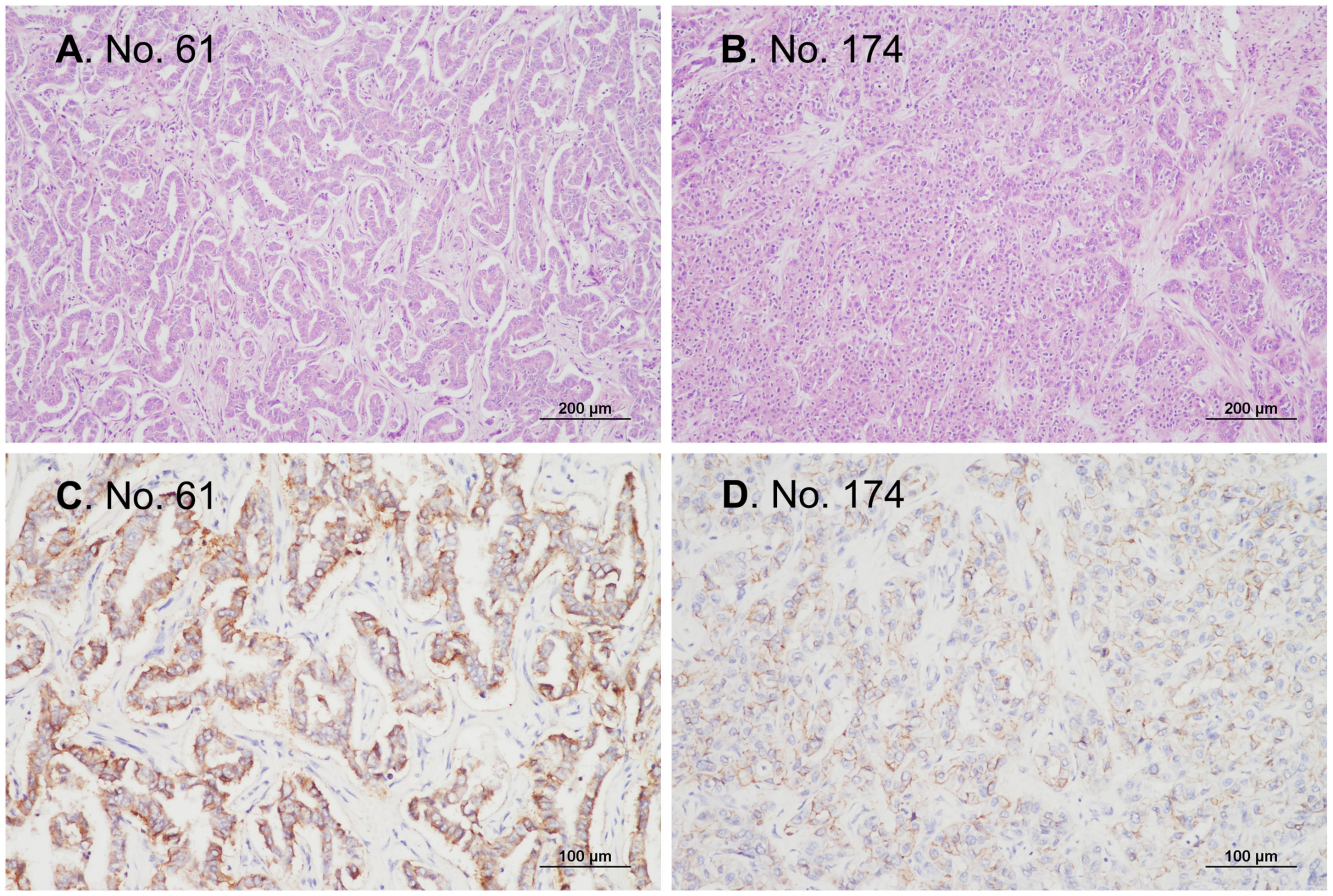
Representative microphotographs of tissues from patients with *IDH*-mutant cholangiocarcinoma. All patients with *IDH*-mutant cholangiocarcinoma in the current study were patients with conventional adenocarcinomas that were immunopositive for CD56. **(A,B)** H&E staining, original magnification ×100. **(C,D)** Immunohistochemistry, original magnification ×200.

In total, samples from 195 patients with CC and 11 patients with BilIN were screened for the presence of *IDH* mutations. *IDH* mutations were detected in six patients with CC by PNA clamping; further validation with NGS confirmed the presence of these mutations. Five patients harbored mutations in codon 132 of *IDH1* (R132C in four patients and R132G in one patient), while one patient had mutations in codon 172 of IDH2 (R172W) (Figure 2). No *IDH* mutations were identified in patients with BilIN. In patients with CC, the presence of *IDH* mutations was significantly associated with larger tumor size (>4 cm; *P* = 0.043). Although patients harboring *IDH* mutations tended to have histologically low-grade tumors, the association between *IDH* mutations and tumor grade was not statistically significant (*P* = 0.082). Furthermore, *IDH* mutations were significantly associated with normal CA19-9 serum levels (*P* = 0.005). There were no significant relationships between *IDH* mutations and the following factors: age, sex, tumor location, histologic type, presence of parasites or hepatitis, pathologic stage, metastasis, or surgical margin status (Table 1). Additionally, there were no significant association between *IDH* mutations and these additional factors: the presence of bile duct stones, inflammation, cirrhosis, BilIN, or intraductal papillary neoplasms. This absence of an association was also true regarding histopathological features of the tumor, including histologic components of mucinous, signet ring, or sarcomatoid cells (all *P* > 0.05, Table 2). In addition, CD56 immunohistochemistry was performed using tissues from six patients with *IDH* mutations; patchy and focal CD56 expression was observed in all tumors with *IDH* mutations (Figure 3). There were no notable histopathological differences among different *IDH1* or *IDH2* mutations.

**Table 2:** Associations between *IDH* mutations and histopathological features.

**Histological features**		**IDH mutation**		***P*-value**
		**Present**	**Absent**	
Mucinous or signet ring cell	Absent	6	185	0.719
	Present	0	4	
Sarcomatoid component	Absent	6	181	0.607
	Present	0	8	
Combined HCC/CC	No	6	164	0.340
	Yes	0	25	
BilIN-low grade	Absent	6	172	0.442
	Present	0	17	
BilIN-high grade	Absent	6	173	0.457
	Present	0	16	
Intraductal papillary neoplasm	Absent	6	172	0.442
	Present	0	17	
Bile duct stone	Absent	6	180	0.584
	Present	0	9	
Periductal inflammation	Minimal to mild	6	171	0.428
	Moderate to severe	0	18	
Cirrhosis	Absent	6	164	0.340
	Present	0	25	

*IDH, isocitrate dehydrogenase; CC, cholangiocarcinoma; HCC, hepatocellular-cholangiocarcinoma; BilIN, biliary intraepithelial neoplasia*.

### Identification of DFS and OS Predictors in Patients With CC

**Figure 4:**
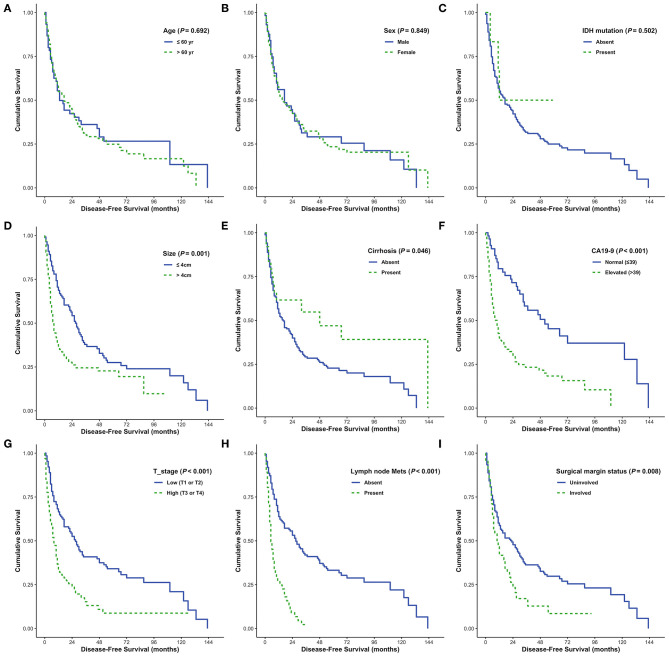
Disease-free survival (DFS) analyses using the Kaplan-Meier estimator and log-rank test were performed according to age **(A)**, sex **(B)**, IDH mutations **(C)**, tumor size **(D)**, cirrhosis **(E)**, CA19-9 **(F)**, T stage **(G)**, lymph node metastasis **(H)**, and surgical margin status **(I)**.

DFS was analyzed in relation to clinical variables. Univariate and multivariate analyses revealed significant effects of the presence of cirrhosis, lymph node metastasis, surgical margin status, or serum CA 19-9 levels on patient survival (all *P* < 0.05; Figure 4). Although univariate analysis revealed that smaller tumor size (*P* = 0.001) and low pathological T stage (*P* < 0.001) were significantly associated with prolonged DFS, neither of these was identified as an independent prognostic factor by multivariate analysis (*P* = 0.291 and *P* = 0.355, respectively). Moreover, sex and enhanced periductal inflammation were significantly associated with DFS in multivariate analysis (*P* = 0.013 and *P* = 0.001, respectively). Neither univariate nor multivariate analysis identified *IDH* mutations as significant prognostic factors in CC (both *P* > 0.05, Table 3).

**Table 3:** Univariate and multivariate analysis for progression-free survival predictors in 195 patients with cholangiocarcinoma.

**Variables**		**No**.	**Mean survival** **(months)**	***P*-value** **(univariate)**	***P*-value** **(multivariate)^†^**	**Hazard ratio**	**95% confidence interval**	
Age	≤ 60 years	60	44.4	0.692	0.837	1	0.644	1.720
	>60 years	135	38.8			1.053		
Sex	Male	138	41.6	0.849	0.013	1.841	1.136	2.981
	Female	57	41.7			1		
IDH mutation	Absent	189	40.6	0.502	0.748	1	0.18	3.462
	Present	6	34.0			0.784		
Size	≤ 4 cm	111	47.9	0.001	0.291	1	0.794	2.156
	>4 cm	84	27.2			1.309		
Location	Intrahepatic	168	44.0	0.300	0.549	1	0.429	1.569
	Extrahepatic	27	26.6			0.82		
Histological grade	Low-grade	131	40.0	0.794	0.804	1	0.66	1.758
	High-grade	64	42.8			1.065		
Periductal inflammation	Minimal to mild	177	40.3	0.448	0.001	1	0.01	0.551
	Moderate to severe	18	38.3			0.224		
Cirrhosis	Absent	170	37.6	0.046	0.018	1	0.091	0.802
	Present	25	69.1			0.27		
CA19-9	Normal (≤ 39)	56	68.5	<0.001	0.003	1	1.22	3.598
	Elevated (>39)	84	27.1			2.156		
T stage	Low (T1 or T2)	128	50.5	<0.001	0.355	1	0.76	2.026
	High (T3 or T4)	67	21.7			1.254		
Lymph node mets	Absent	149	50.8	<0.001	<0.001	1	2.55	7.614
	Present	46	9.4			4.445		
Distant mets	Absent	191	41.4	0.201	0.422	1	0.125	2.394
	Present	4	7.3			0.546		
Surgical margin status	Uninvolved	152	45.6	0.008	0.050	1	1.000	2.852
	Involved	43	20.6			1.688		

*IDH, isocitrate dehydrogenase*.

† *Cox proportional hazards model for multivariate analysis*.

**Figure 5:**
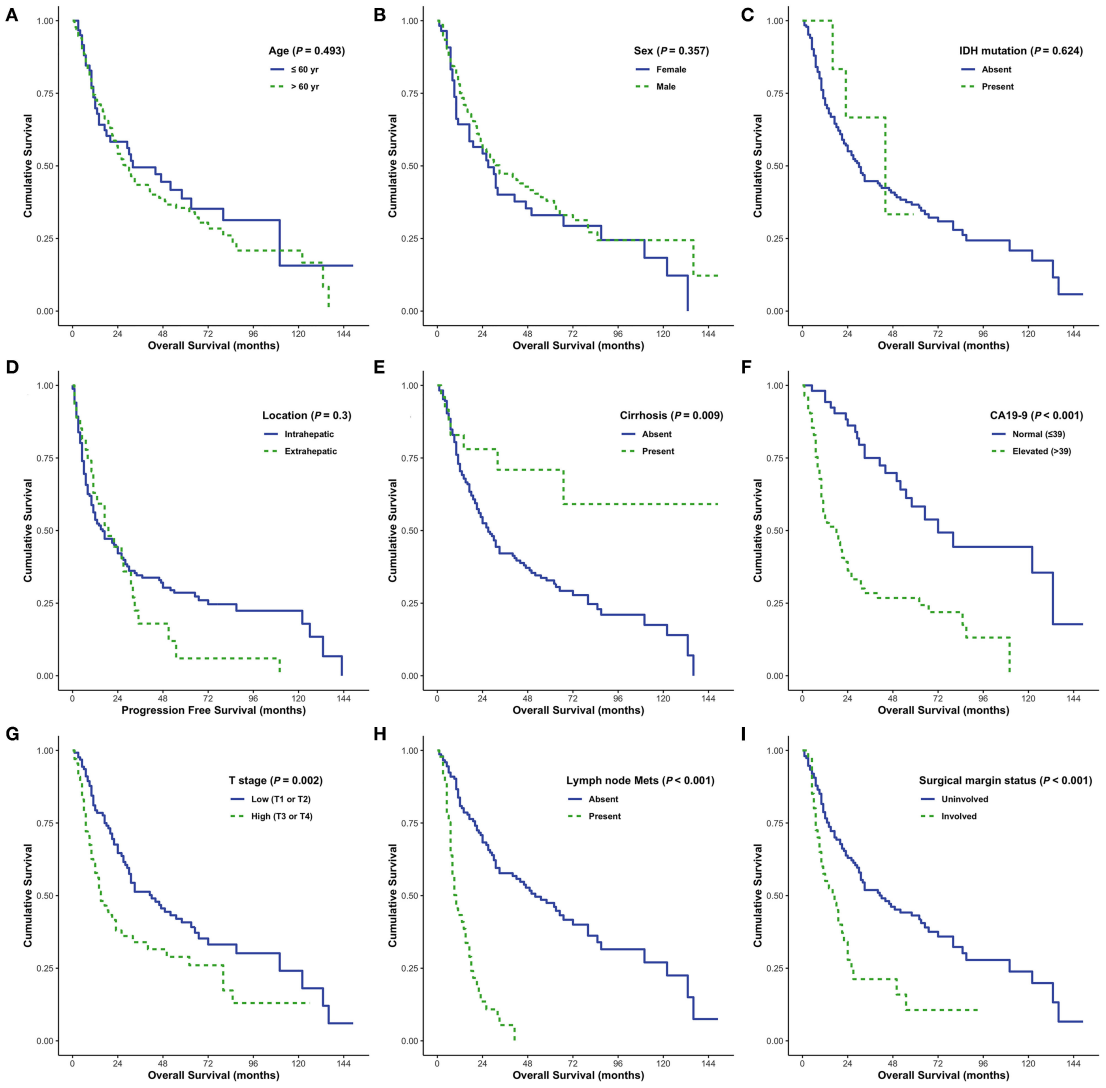
Overall survival (OS) analyses using the Kaplan–Meier estimator and log-rank test were performed according to age **(A)**, sex **(B)**, IDH mutations **(C)**, tumor size **(D)**, cirrhosis **(E)**, CA19-9 **(F)**, T stage **(G)**, lymph node metastasis **(H)**, and surgical margin status **(I)**.

Elevated serum CA19-9 levels, lymph node metastasis, and positive surgical margins were independent predictors of poor OS in patients with CC (all *P* < 0.05; Figure 5). Moreover, cirrhosis was associated with prolonged survival in both univariate and multivariate analyses (*P* = 0.009 and *P* = 0.072, respectively). Intrahepatic CC and absence of distant metastases were associated with prolonged survival in univariate analysis (*P* = 0.064 and *P* = 0.069, respectively); however, no significant association was identified by multivariate analysis. Enhanced periductal inflammation showed a significant association with improved OS in multivariate analysis (*P* = 0.012). Age, sex, tumor size, and histological grade were not significantly associated with OS. Furthermore, no significant associations were observed between the presence of *IDH* mutations and OS (both *P* > 0.05, Table 4).

**Table 4:** Univariate and multivariate analysis for overall survival predictors in 195 patients with cholangiocarcinoma.

**Variables**		**No**.	**Mean survival** **(months)**	***P*-value** **(univariate)**	***P*-value** **(multivariate)^†^**	**Hazard ratio**	**95% confidence interval**	
Age	≤ 60 years	60	58.3	0.493	0.326	1	0.459	1.295
	>60 years	135	51.0			0.771		
Sex	Male	138	49.2	0.357	0.402	1.231	0.757	2.005
	Female	57	57.2			1		
IDH mutation	Absent	189	53.6	0.624	0.836	1	0.15	3.914
	Present	6	40.1			0.851		
Size	≤ 4 cm	111	58.3	0.108	0.624	1	0.659	2.003
	>4 cm	84	45.0			1.149		
Location	Intrahepatic	168	57.8	0.064	0.689	1	0.443	1.714
	Extrahepatic	27	34.3			0.871		
Histological grade	Low-grade	131	50.3	0.417	0.290	1	0.42	1.294
	High-grade	64	62.6			0.739		
Periductal inflammation	Minimal to mild	177	53.6	0.639	0.012	1	0.17	0.772
	Moderate to severe	18	45.3			0.313		
Cirrhosis	Absent	170	49.0	0.009	0.072	1	0.058	1.131
	Present	25	99.8			0.256		
CA19-9	Normal (≤ 39)	56	85.0	<0.001	0.001	1	1.56	5.020
	Elevated (>39)	84	34.8			2.794		
T stage	Low (T1 or T2)	128	60.5	0.002	0.635	1	0.55	1.499
	High (T3 or T4)	67	38.4			0.879		
Lymph node mets	Absent	149	65.9	<0.001	<0.001	1	2.73	8.584
	Present	46	13.7			4.896		
Distant mets	Absent	191	54.3	0.069	0.298	1	0.495	9.919
	Present	4	13.5			2.216		
Surgical margin status	Uninvolved	152	60.1	<0.001	0.008	1	1.221	3.765
	Involved	43	25.8			2.144		

*IDH, isocitrate dehydrogenase*.

† *Cox proportional hazards model for multivariate analysis*.

## Discussion

Despite recent improvements in understanding the molecular mechanisms underlying cancer, little is known regarding genomics in CC. The genomic profiling of CC and analysis of *IDH* mutations, in particular, have gained increasing interest in recent years. *IDH1* and *IDH2* exhibit high sequence similarity and are often present in various malignancies, including glioma, leukemia, and cartilaginous tumors (16). *IDH1* mutations have been identified in 9.2% (191 of 2,079) of biliary tract tumors in the COSMIC database (as of March 2020). In the present study, we identified *IDH1* and *IDH2* mutations in 3.1% (six of 195) of patients with CC; hence, the incidence of *IDH* mutations in our cohort was relatively low, compared to the incidences in previous reports (5–7, 9, 17). However, these results were consistent with the findings of previous studies in Asian populations, which reported low frequencies of *IDH* mutations. Because the vast majority of data regarding *IDH* mutations in CC are derived from studies in non-Asian populations, differences in patient ethnicities among studies may contribute to discrepancies in *IDH* mutation frequencies. Risk factors associated with CC include liver flukes and chronic viral hepatitis, which are prevalent in Asian countries; thus, differences in risk factor distributions might partly explain the differences in *IDH* mutation prevalences in CC among individuals of different ethnicities.

Moreover, *C. sinensis* and *O. viverrini* infestations are strongly associated with CC development. Chan-On et al. reported that *IDH1* and *IDH2* mutations were more frequent in non-*O. viverrini* CC, compared to O. viverrini-associated CC (10). However, we were unable to reproduce the previously reported associations between *IDH* mutations and parasite infestation. Future large cohort and multi-ethnic studies are needed to confirm the relationships between liver fluke infestation and *IDH* mutations.

Types of IDH mutations vary significantly among tumor types. *IDH1* mutations in codon R132 are the predominant *IDH* mutations in brain tumors; these mutations are functionally similar to R172 mutations in *IDH2*. *IDH1* R132H mutations represent ~90% of all glioma-associated *IDH* mutations; *IDH1* R132C, R132S, R132G, and R132L, as well as *IDH2* R172K, R172M, and R172W represent the remaining 10%. Unlike glioma, most previously reported *IDH* mutations in CC occurred in codon R132C of *IDH1*. Our study confirmed the presence of *IDH1* and *IDH2* mutations in CC. Among the six resected CC specimens harboring *IDH1* or *IDH2* mutations, mutations in codon R132C and R132G of *IDH1* were detected in specimens from five patients, while a specimen from the remaining patient had mutations in codon R172W. *IDH1* mutations were more frequent than *IDH2* mutations in CC, and the majority of *IDH1* mutations were present in codon R132C, consistent with the findings of previous studies. However, unlike glioma, R132H mutations were not observed in our cohort. *IDH* mutations in glioma typically accumulate in lower-grade gliomas early during tumor initiation and are maintained throughout progression to high-grade malignancy (18). To assess the prevalence of *IDH* mutations early during CC development, we analyzed *IDH* mutations in samples from patients with BilIN, which constitute precursor lesions of CC. However, no IDH mutations were detected in BilIN specimens.

*IDH* mutations are found in ~20% of patients with intrahepatic CC. Borger et al. presumed that *IDH1* mutations represented a molecular feature of CC of intrahepatic origin (4). Kipp et al. also found that *IDH* mutations were more frequent in intrahepatic CC than in extrahepatic CC; moreover, *IDH* mutations were associated with clear cell lesions and poorly differentiated histology (7). In the present study, we found that all *IDH*-mutant CC samples were CD56-positive; thus, it is likely that these tumors represented the small duct type of intrahepatic CC (19). In CC, conflicting data exist regarding the prevalence and clinical significance of *IDH* mutations. Most previous studies focused on *IDH1* genetic profiling in patients with intrahepatic CC; conversely, the present study involved analysis of mutations in both *IDH1* and *IDH2* in a large cohort of patients with diverse types of CC. We found that *IDH* mutations tended to be associated with low-grade histology, although this relationship was not statistically significant (*P* = 0.082). No other histological types or features were associated with *IDH1* or *IDH2* mutations. Consistent with the results of prior studies, we found that all instances of *IDH*-mutant CC originated in the intrahepatic area. Given that the majority of CC samples were of intrahepatic origin and were acquired during surgical resection of the liver, the frequency of *IDH* mutations might have been skewed.

The relationships between *IDH* mutations and prognosis have been investigated in various tumors. *IDH* mutations were associated with favorable outcomes in patients with glioma (20). In contrast, worse OS or no impact on prognosis was observed in patients with acute myeloid leukemia (21). In a cohort of 326 patients with resected intrahepatic CC, Wang et al. found that patients with *IDH* mutations exhibited prolonged DFS and OS (5). Another study showed that *IDH1* mutations in intrahepatic CC were associated with favorable prognosis, smaller tumor size, lower serum CA19-9 levels, and lower TNM stage. Furthermore, *IDH1* mutations reportedly affected growth inhibition in intrahepatic CC by suppressing AKT signaling, both *in vivo* and *in vitro* (8). In contrast, analysis of a cohort of 32 patients with *IDH* mutant or *IDH* wild-type intrahepatic CC revealed shorter median OS in patients with *IDH* mutations (3). However, more patients with *IDH* mutations had stage IV disease (50%), compared to patients with *IDH* wild-type (15%); this difference might have affected the survival rate and prognosis. Zhu et al. detected *IDH* mutations in 20% of the patients (15.5% *IDH1* and 4.5% *IDH2*) in their study of 200 patients with resected intrahepatic CC (6); no significant differences in DFS or OS were reported among patients with different *IDH* mutation statuses. Similarly, Goyal et al. found no association between *IDH* mutations and prognosis in patients with intrahepatic CC; however, patients with *IDH* mutations had lower serum CA19-9 levels at presentation (9). We found that *IDH* mutations were more frequent (5/6) in patients with larger tumor size (>4 cm). This finding was consistent with the results described by Goyal et al., who reported that *IDH* mutations were significantly associated with physiological serum CA19-9 levels. In addition, they observed no associations between mutational status and long-term outcomes. We also found no significant associations between *IDH* mutations and OS or DFS in patients with CC.

There were some limitations in this study. First, the number of extrahepatic cases was relatively small because patients who underwent liver resection were enrolled in this study. Second, the study cohort was from a single institution, and the results might have been influenced by the regional characteristics of the patients. Nevertheless, this study included a large Asian cohort. Data regarding genetic variation in CC are primarily derived from studies in Western cohorts, which have a relatively low incidence of hepatobiliary cancer. Therefore, analysis of genetic variation in Asian patients with CC is crucial. The identification of genetic characteristics associated with risk factors for CC, one of the most intractable cancers with poor prognosis, will foster the development of more effective treatment strategies.

## Data Availability Statement

The datasets presented in this study can be found in online repositories. The names of the repository/repositories and accession number(s) can be found below: https://www.ebi.ac.uk/ena/browser/view/PRJEB38130.

## Ethics Statement

The studies involving human participants were reviewed and approved by Institutional Review Board of the Chonnam National University Hwasun Hospital (CNUHH-2019-213). The patients/participants provided their written informed consent to participate in this study.

## Author Contributions

K-HL and J-HL designed this study. EW and YL performed the experiments. NK and M-GN drafted the manuscript. M-GN and J-HK performed data analysis. YH and K-SM collected clinical data. NK and K-HL performed the pathological examination. K-HL and M-GN performed statistical analyses. K-SM and J-HL assisted with manuscript preparation and data analysis. All authors read and approved the final manuscript.

## Conflict of Interest

The authors declare that the research was conducted in the absence of any commercial or financial relationships that could be construed as a potential conflict of interest.
