# Supplementary material for: Frequency and Prognostic Value of IDH Mutations in Korean Patients With Cholangiocarcinoma
Source: Front Oncol. 2020 Aug 18;10:1514. doi: 10.3389/fonc.2020.01514 (PMC7461833; doi:10.3389/fonc.2020.01514)
Supplement: Supplementary file 1 [file Table_1.DOCX]

Supplementary Material

**Supplementary Table S1**. IDH1 and IDH2 mutations that can be detected with PNAClamp™ IDH Mutation detection kit

| Gene | Exon | Mutation | Base change |
| --- | --- | --- | --- |
| IDH1 | Exon 4 | R132G | 394C>G |
|  |  | R132C | 394C>T |
|  |  | R132S | 394C>A |
|  |  | R132L | 394C>T |
|  |  | R132H | 394C>A |
| IDH2 | Exon 4 | I139F | 415A>T |
|  |  | R140W | 418C>T |
|  |  | R140Q | 419G>A |
|  |  | R140L | 419G>T |
|  |  | G171D | 512G>A |
|  |  | R172G | 514A>G |
|  |  | R172W | 514A>T |
|  |  | R172K | 515G>A |
|  |  | R172M | 515G>T |
|  |  | R172S | 516G>C |
|  |  | R172S | 516G>T |
